# Non-coding RNA and reprogrammed mitochondrial metabolism in genitourinary cancer

**DOI:** 10.3389/fgene.2024.1364389

**Published:** 2024-03-13

**Authors:** Sandiya Thirunavukkarasu, Shouryarudra Banerjee, Ishaq Tantray, Rani Ojha

**Affiliations:** ^1^ Department of Urology, Post Graduate Institute of Medical Education and Research, Chandigarh, India; ^2^ InventX Scientia, Kashmir, India; ^3^ Department of Pathology, School of Medicine, Stanford University, Stanford, CA, United States

**Keywords:** non-coding RNAs, mitochondria, metabolism, cancer, therapy, resistance

## Abstract

Non-coding ribonucleic acids (ncRNAs) have been recently shown to contribute to tumorigenesis by mediating changes in metabolism. ncRNAs act as key molecules in metabolic pathways regulation. The dysregulation of ncRNAs during cancer progression contributes to altered metabolic phenotypes leading to reprogrammed metabolism. Since ncRNAs affect different tumor processes by regulating mitochondrial dynamics and metabolism, in the future ncRNAs can be exploited in disease detection, diagnosis, treatment, and resistance. The purpose of this review is to highlight the role of ncRNAs in mitochondrial metabolic reprogramming and to relate their therapeutic potential in the management of genitourinary cancer.

## 1 Introduction

Energy metabolism is of great importance in the metabolic reprogramming of cancer, where the metabolic flux is increased in the tumor cells compared to the precursor tissue of origin. This ‘energy-dependent metabolic flux’ is powered by mitochondrial metabolic reprogramming which activates various oncogenic signaling pathways (Scheid et al., 2021). The majority of cellular energy is provided through the mitochondrial metabolism. The cancer cells rely only on glycolysis to meet their bioenergetic demands, but they still are dependent on some of the mitochondrial electron transport (mETC) byproducts for effective cell proliferation. This suggests that respiratory defects or dysfunction in mitochondrial dynamics could be the primary cause of cancer, as observed by Otto Warburg in the ‘Warburg effect (Cantor and Sabatini, 2012; Ward and Thompson, 2012; Chen et al., 2023; Kaur et al., 2023; Wang and Patti, 2023). These discoveries emphasize the impact of mitochondrial function in cancer progression and could have significant implications for cancer treatment. Additionally, mitochondria are linked to redox regulation, cell signaling, apoptosis, and cell function and fate (DeBerardinis and Chandel, 2016; Chen et al., 2023). Furthermore, various studies revealed that mitochondrial metabolic reprogramming is related to the development of genitourinary cancer such as bladder cancer, prostate cancer, and kidney cancer. Additionally, genitourinary cancer is characterized by the upregulation of several oncometabolites, such as glucose, glutamine, succinate, fumarate, malate, lactate, and itaconate (Sullivan et al., 2013; Shim et al., 2014; Yong et al., 2020; Delkov et al., 2022). Going forward, we predict that mitochondrial oncometabolite will continue to shed new light on disease progression. Therefore, it is essential to review and understand the crosstalk between mitochondrial metabolic reprogramming and genitourinary cancer for effective clinical management.

The understanding of RNA biology has improved significantly over the last decade. In the human genome, about 80% is transcribed to RNA, however, there are significant untranslated RNAs called non-coding RNAs (ncRNAs). They are mainly categorized into two classes: small ncRNAs microRNAs (miRNAs) and long non-coding RNAs (lncRNAs). Another known ncRNA is circular RNAs (circRNAs), which have also been known as a critical regulator of gene expression (Mattick and Makunin, 2006; Slack and Chinnaiyan, 2019). Recent research has established a link between ncRNAs and mitochondrial processes such as energy metabolism, oxidative phosphorylation, redox regulation, gene expression, protein transport, and mitochondrial proteome homeostasis (Table 1). The mitochondrial ncRNAs (mt-ncRNAs) can be mitochondrial encoded which can be generated inside the mitochondria or nuclear-encoded which can be imported into mitochondria (Villegas et al., 2007; Liu and Shan, 2021; Gallo Cantafio et al., 2023). Understanding the relationship involving ncRNAs and mitochondrial metabolism not only provides deeper insights into the mechanisms but also offers the development of new targeted anticancer therapeutics. Notably, some ncRNAs involved in cellular signaling pathways of genitourinary cancer, also have significant associations with mitochondrial functions and metabolism. Therefore, comprehensive knowledge of the interplay among ncRNAs and mitochondrial metabolism is fundamental for effective genitourinary cancer diagnosis and treatment.

**Table 1:** ncRNAs and mitochondrial metabolism crosstal

NcRNA	Involved Process	Type	Target	Molecular Effect
GAS5	TCA	LncRNA	MDH2	Promoting the association of FH-MDH2-CS
MecciND1	Mitochondrial DNA replication	CircRNA	RPA32/70	Enhancing the mitochondrial localization of RPA32/70
SAMMSON	Mitochondrial translation	LncRNA	P32	Enhancing the mitochondrial localization of P32
LncFAO	β-oxidation	LncRNA	HADHB	Increasing of HADHB level
SCAR	MPTP opening	CircRNA	ATP5B	Inhibiting the interaction between ATP5B and CypD
CircSmad4		CircRNA	VCP	Enhancing the mitochondrial localization of VCP
miR-1	OXPHOS	miRNA	ND1 and COX1 mRNA	Enhancing translation of ND1 and COX1
miR-21		miRNA	CYTB mRNA	Enhancing translation of CYTB
miR-181c		miRNA	COX1 mRNA	Decreasing protein level of COX1
miR-378		miRNA	ATP6 mRNA	Decreasing protein level of ATP6
let-7a		miRNA	ND4 mRNA	Decreasing protein level of ND4
miR-2392		miRNA	Mitochondria DNA	Enhancing transcription of mitochondrial DNA
CircPUM1		CircRNA	UQCRC2	Promoting the association of UQCRC1 and 2
MALAT1		LncRNA	Mitochondria DNA	Inhibiting methylation of mitochondrial DNA

### 1.1 Genitourinary cancer

Genitourinary cancer (GC) engirds a group of heterogeneous cancers about three major organs kidney (2.3%), bladder (3.2%), and prostate (7.8%). The major histological subtypes of this cancer include renal cell carcinoma, urothelial carcinoma, and prostate cancer (Zarrabi et al., 2019; Sung et al., 2021). Renal cell carcinoma (RCC) is classified as clear cell RCC which is among the 80% diagnosed and the other 20% are nonclear cell RCC (Zarrabi et al., 2019; Riscal et al., 2021). Unfortunately, the tumor proved resistant to anticancer therapies. As a result, RCC has been challenging to treat (De Meerleer et al., 2014; Ross and Jones, 2017; Linehan and Ricketts, 2019).

Urothelial carcinoma is the most prevalent type of urinary bladder cancer. Its tumorgenicity can be presented by 70%–75% of non-muscle-invasive bladder cancer (NMIBC) and 30% of the muscle-invasive bladder (MIBC). MIBC has a high mortality rate compared to NIMBC as it has a limited metastatic disease potential, though it depicts a high recurrence rate (Cheng et al., 2009; Lavallee et al., 2021; Huang et al., 2022).

Prostate cancer (PC) is the most frequent cancer in men. The risk of developing PC is very frequent. The treatment of PC contingents on the stages of the disease, histological grade, and serum prostate-specific antigen level. Radical prostatectomy is regularly used to treat localized PC. However, the recurrence rate (27%–53%) is very high (Hu et al., 2009; Vikramdeo et al., 2023).

### 1.2 Non-coding RNAs

The non-coding RNAs (ncRNAs) are less frequently expressed than the protein-coding genes, where their characteristic functional structures are well conserved across evolutionary timescales. It is well-established that ncRNAs function as both tumor enhancers and tumor suppressors in nearly all types of cancer (Mattick and Makunin, 2006; Tantray et al., 2023). Despite these expression patterns, ncRNAs are precisely tuned to specific tissues or cancer types, regulating complex mechanisms (Table 2). Thus, they establish an elaborate network of interactions that contribute to cancer development and progression (Grillone et al., 2020). ncRNAs are divided into long non-coding RNAs, microRNAs, and circularRNAs.

**Table 2:** Overview of ncRNA roles in cancer metabolism.

ncRNA	Function of ncRNA in cancer	Dysregulated in cancer	Mechanisms of action
HOTAIR	Tumor promoter	Endometrial, lung, ovarian, prostate, thyroid	Interacts with PRC2 to methylate and silence tumor suppressor genes
BRAFP1		Lymphoma	Activates BRAF
NANOG		Breast, colorectal, hepatocellular, leukemia, lung, pancreatic, prostate	Sustains cell renewal and confers stem cell-like properties.
Oct-04		Liver, lung, pancreas	Sustains cell renewal and confers stem cell-like properties.
circPRKCI		Glioma, lung	Promotes proliferation and migration by sponging miR-545
circHIPK3		Breast, colorectal, gallbladder, gastric, ovarian	Promotes cancer growth and metastasis by sponging miR-7, miR-193a
MYLK		Lung cancer	Promotes glycolysis and proliferation
LDLRAD		Lung cancer	Promotes proliferation and survival
517		Lung cancer	Promotes glycolysis and clonogenicity
piR-651		Breast, colorectal, head and neck, leukemia, lung, lymphoma, pancreatic, renal	Functions with C-MYC and transcriptional regulation, regulates proliferation, apoptosis, angiogenesis
miR-518b, miR-629		Lung Cancer	Promotes proliferation metastasis
miR-141		Prostate cancer	Promotes prolifera
miR-1274a, miR-592		Colon cancer	Promotes proliferation, meta and clonogen
miR15/16		Leukemia	Sustains stemnes
MEG3	Tumor suppressor	Breast, colorectal, gastric, liver, lung, ovarian, prostate	Regulates proliferation, angiogenesis, epithelial-to- mesenchymal transition, drug sensitivity
PTENP1		Breast, gastri represses expression of k-Ras c, prostate, Renal	Sponges microRNAs that target PTEN
miR-30, miR-140, miR-143, miR-600, miR-7		Breast cancer	Promotes apopt
let-7, miR- 200a, miR- 190b		Lung cancer	Represses expression of k-Ras, inhibits stemness and cell gro
miR-145, miR-34		Prostate cancer	Inhibits proliferation and inv reduced stemness
MALAT1	Tumor promoter and tumor suppressor	Breast, endometrial, lung, ovarian, prostate, thyroid	Alternative splicing, metastasis
H19		Bladder, breast, colorectal, endometrial, ovarian, prostate	Induces cell survival pathways in response to stress, epithelial-to- mesenchymal transition
piR-823		Colorectal, esophageal, gastric, Breast, Lung	Affects cell growth, metastasis, DNA methylation, apoptosis, transcriptional activity
piR-932		Breast, endometrial, glioblastoma, hepatocellular, pancreatic, prostate, thyroid	Targets tumor suppressors. Induces cell proliferation, drug resistance

Long non-coding RNAs (lncRNAs) are generally about 200 nucleotides to 100 kilobases. Genomic regions transcribed into certain low-level lncRNAs have fewer exons, known as long intergenic RNAs (lincRNAs) (Ransohoff et al., 2018; Gallo Cantafio et al., 2023). There are over 5,400 to 10,000 lncRNA generated from various DNA elements in the genome. LncRNAs are implicated in the regulation of embryonic stem cell differentiation, as well as being involved in various disease progression (Villegas et al., 2007; Mattick et al., 2023). The lncRNA expression is more specific to cell and tissue type compared to protein-coding genes. The sequence similarity of lncRNA is conserved in secondary structures (Hung et al., 2014; Xu et al., 2021; Mattick et al., 2023). lncRNAs have the potential to form complex three-dimensional structures due to their long length and can contain multiple structural or functional domains. They also have a high number of protein-binding sites for the multimerization of proteins or scaffolding for the assembly of large multimeric proteins (Ma et al., 2013). The secondary or tertiary structures of lncRNAs play an indispensable role in their interactions with proteins and other nucleic acids to regulate gene expression (Shi et al., 2001; Zampetaki et al., 2018). lncRNAs can regulate gene expression, epigenetic modifications, transcription, post-transcriptional activity, and metabolic function. Additionally, LncRNAs indirectly modulate gene expression via RNA-binding protein partners or miRNAs (Olgun et al., 2018; Li et al., 2020).

MicroRNAs (miRNAs) are short ncRNA molecules (∼22 nucleotides) (O’Brien et al., 2018). miRNAs typically interact with the promoter region, 3′ UTR & 5′ UTR region, coding sequence, and gene promoters, to suppress the expression of the target gene (Gu et al., 2009). The miRNAs are capable of activating gene expression by two mechanisms, via inhibiting translation or by degradation of complementary mRNA. miRNAs are transported or exported within the intracellular compartments to regulate cell fate by controlling transcription and translational activity (Peng and Croce, 2016).

Single-stranded, covalently closed circRNAs possess a unique structure with a longer half-life and have recently been involved in various diseases including cancer (Zhou et al., 2020; Raza et al., 2022). Additionally, circRNAs were shown to act as miRNA sponges (Bosson et al., 2014). However, the role of circRNAs in physiological or pathological conditions remains poorly understood.

## 2 Mitochondria-encoded non-coding RNAs

The mitochondrial genome contains numerous ncRNAs, such as mitochondrial transfer RNAs (mt-tRNAs), mitochondrial long non-coding transfer RNAs (mt-lncRNAs), mitochondrial miRNAs (mt-miRNAs), mitochondria-encoded circRNAs (mtcciRNAs), an antisense noncoding mitochondrial RNAs (ASncmtRNAs), and mitochondrial double-stranded RNAs (mt-dsRNAs). These mitochondrial non-coding RNAs (mt-ncRNAs) are essential in regulating different physiological and pathological processes (Ren et al., 2023). Several hereditary human diseases are caused by mutations in mt-tRNAs, while other mt-ncRNAs are associated with metabolic disorders and cancers such as breast cancer, hepatocellular carcinoma, leukemia, and other genitourinary cancers (Villegas et al., 2007; Slack and Chinnaiyan, 2019). The revolutionary tools in mitochondrial biology, such as mitochondrial genome editing, are set to provide researchers with a better understanding of the biogenesis, metabolism, and functions of mt-ncRNAs (Liu and Shan, 2021).

From the mitochondrial genome, several lncRNAs such as lncND5/6, and lncCyt b, have been identified. It is believed that these lncRNAs have an important functional role in stabilizing the mRNAs of ND5, ND6, and Cyt b (Dong et al., 2017). It is suggested that these lncRNAs regulate mRNA expression by forming intermolecular duplexes with their complementary mRNAs (Mercer et al., 2011; Rackham et al., 2011). A study by Dasgupta et al. (2008) established that the upregulation of mtCytb in the MB49 bladder cancer cell line increased oxidative stress, mitochondrial metabolism, and lactate production, which promote tumor growth by increasing the NF-κB2 signaling pathway. These findings suggest that mutations in mitochondrial-encoded proteins play an oncogenic role in bladder cancer cells.

A study by Dhir et al. (2018) showed that HeLa cells have unstable mt-dsRNA. The RNA degradosome present in the mitochondria, comprising small unilamellar vesicles 3 (SUV3) and polyribonucleotide 1 (PNPT1) components, rapidly breaks down the light-strand transcript of mtDNA. This degradosome strictly monitors the unstable mt-dsRNAs. When SUV3 or PNPase is silenced, it results in a significant build-up of mt-dsRNAs. Arnaiz et al. (2021) showed that hypoxia leads to a decrease in mt-dsRNA production during chemotherapy via inhibition of interferon β production.

Mitochondria-encoded circRNAs (mtcciRNAs) were localized inside the mitochondria and in the cytosol. Two mtcciRNAs, mtcciND1, and mtcciND5, demonstrated to have an essential role in the physiological functions of mitochondria. mtcciND1 binds to the replication proteins (RPA1 and RPA2) involved in mtDNA replication. The expression level of mtcciND1 is positively highly correlated with the levels of mitochondrial RPA proteins and mtDNA copy numbers (Vartak et al., 2015). mtcciND5 interacted with three heterogeneous nuclear ribonucleoproteins (hnRNPs), hnRNPA1/2B1/3, and promoted their mitochondrial importation (Liu et al., 2019; Liu et al., 2020). mtcciND1 and mtcciND5 interact with translocase of the outer membrane of mitochondria 40 (TOM40) and polynucleotide phosphorylase (PNPASE), to act as molecular chaperones (Gabriel et al., 2003; Wang et al., 2010; Wang et al., 2012). An antisense mtcciSCAR from the locus Cytochrome c oxidase 2 (COX2) was found to bind directly to the adenosine triphosphate synthase 5 beta (Hyttinen et al., 2023). The interaction of ATP5B and mtcciSCAR blocks mitochondrial permeability transition pore (mPTP), and therefore reduces mitochondrial ROS (Zhou et al., 2023). Another highly expressed mtcciRNA, mtcciCOX2, was found in chronic lymphocytic leukemia patients (Wu et al., 2020a; Zhao et al., 2020).

Four mt-miRNAs (has-miR-4461/4463/4484/4485) are upregulated in HeLa and HEK cells. Gao et al. identified mt-lncRNAs, hsa-tir-MDL1AS/18 and hsa-MDL1, where downregulation of hsa-tir-MDL1AS-18 has been observed in hepatocellular carcinoma tissues, indicating its role in cancer progression (Gao et al., 2018; Pozzi and Dowling, 2019). However, mt-miRNA role in genitourinary cancer is not explored yet.

Silencing of ASncmtRNAs, induced cell death in various cancer cell lines, including prostate, and kidney cancer, making it a promising selective therapy against genitourinary cancer (Liang et al., 2021). An orthotropic murine model showed that ASncmtRNAs silencing induced cell death in mouse renal adenocarcinoma (RenCa) cells, resulting in a delay and even reversal of tumor growth in a RenCa model. This indicates that ASncmtRNAs can be used as a target for therapy in human renal adenocarcinoma (Borgna et al., 2017). In addition, the transfection of Andes-1537S increased cell death and decreased cell metastasis in the UMUC-3 bladder cancer cell line (Borgna et al., 2020).

## 3 Non-coding RNA and mitochondrial metabolism

**FIGURE 1:**
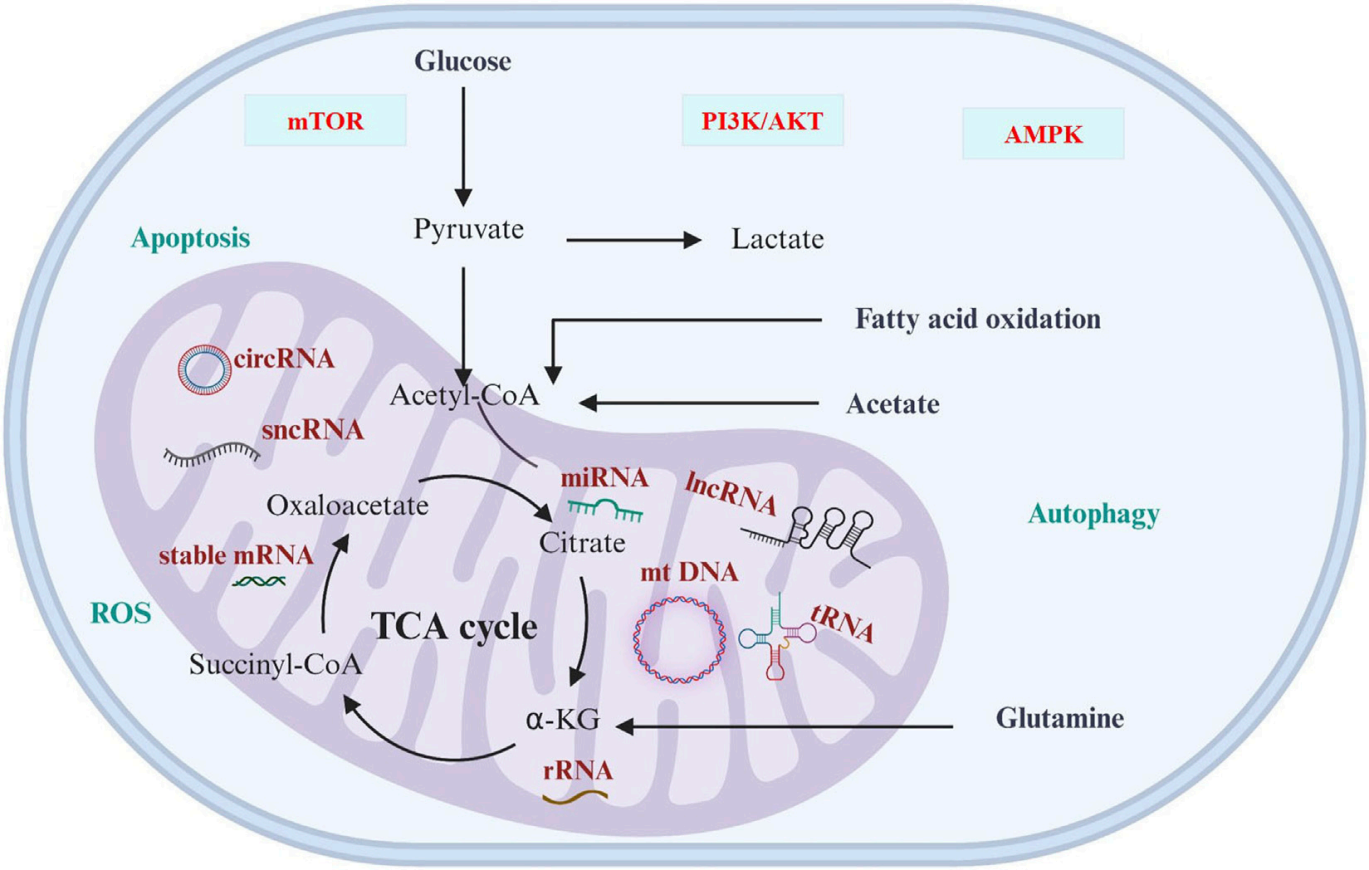
Cancer metabolism and ncRNAs. The significance of ncRNAs in cancer cell metabolic reprogramming is underscored, primarily through modulation of cellular signaling pathways, including AMPK, PI3K/AKT, NFκB, and mTOR. The varied metabolic preferences in cancer present diagnostic and prognostic challenges, influencing predictions of disease progression by impacting redox regulation, apoptosis, as well as cellular function and fate.

**FIGURE 2:**
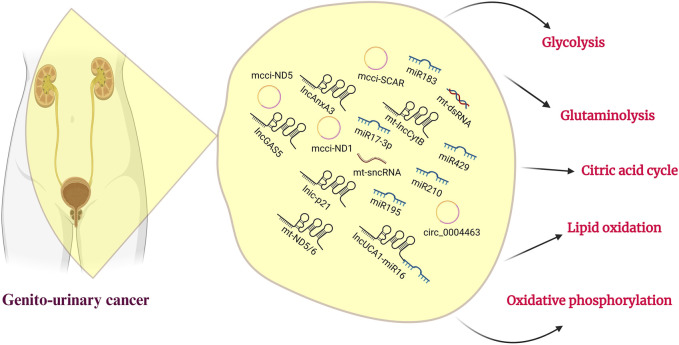
Genitourinary cancer and ncRNAs. A roster of noncoding RNAs and their associated sponges participate in mitochondrial metabolism, with a specific role in regulating glucose, lipids, and amino acid metabolism. This emphasizes their potential for therapeutic targeting in the treatment of genitourinary cancer.

Mitochondria is a central executor of metabolic reprogramming in a variety of cancers, including genitourinary cancer. The main pathways of metabolic reprogramming are glucose metabolism, glutamine metabolism, TCA cycle, and lipid metabolism. These metabolic pathways are regulated by ncRNAs that are linked to cancer progressions (Figure 1; Table 3). This regulation occurs by controlling several cellular signaling pathways, like AMPK, PI3K/AKT, NFκB, and mTOR (You et al., 2023). The metabolic preferences of genitourinary cancer are known to vary, which obstructs the diagnosis and predicts the progression of the disease (Figure 2). However, by identifying and understanding the key mitochondrial alterations associated with them, we can develop diagnostic and prognostic strategies (Konety and Joslyn, 2003; Bismar et al., 2006; Chen et al., 2016).

**Table 3:** The regulatory roles of ncRNA in metabolic pathways in genitourinary cancers.

ncRNA	Description
Glucose Metabolism	
miR-34a/c	Directly target LDH mRNA in pancreatic cancer. Decreased in bladder and prostate cancer tissue. Upregulation inhibits cancer cell growth and viability.
miR-155	Targets C/enhancer binding protein alpha, inhibiting miR143, which inhibits hexokinase 2. Cell-free expression correlates with the stage and grade of bladder cancer and renal cell carcinoma.
mi R-1 24	Regulates genes of both pyruvate kinase M2 (PKM2) and pentose phosphate pathway (PPP) in prostate and bladder cancer. Significantly lower in renal cell carcinoma tissue compared to normal tissue.
LncUCA1	Activates mTOR, inducing signal transducer and activator of transcription 3 protein, and inhibiting miR-143, thereby upregulating hexokinase 2 and glycolysis in bladder cancer.
PCGEM1	Overexpressed in prostate and renal cell carcinoma, promoting glucose uptake for aerobic glycolysis and coupling it with PPP to facilitate nucleotide and lipid biosynthesis.
ln CASC8 c	Reduced in high-grade bladder cancer. Binds to fibroblast growth ftor receptor 1 (FGFR1) and abrogates lactate dehydrogenase phosphorylation, reducing glycolysis and inhibiting bladder cancer cell growth.
lncFILNC1	Knockdown increases c-Myc protein level by FILNC1-AUF1-c- Myc signaling axis under glucose starvation conditions in RCC.
SLC16A1-AS1	Improves glycolysis and mitochondrial respiration by increasing ATP synthesis in bladder cancer, leading to increased proliferation by fatty acid oxidation.
miR-210	Upregulated in RCC predominantly mediated by hypoxia-inducible factor1. Upregulated in blood serum of bladder cancer patients, increases with disease progression. Regulates bladder cancer growth, invasion, and metastasis by targeting FGFRL1. Overexpression is significantly higher in tumor tissues of prostate cancer, correlated with bone metastasis.
Glutamine Metabolism	
miR-23a/b, lncRNA CCAT2, miR-23b*	Concurrent regulation of glutaminase (GLS) by miR23a/b and lncRNA CCAT2. Allele-specific metabolic reprogramming of renal cell carcinoma.
lincRNA-p21	Inhibits bladder cancer proliferation by negatively regulating glutaminase, glutamate, and αketoglutarate expression. Overexpression of glutaminase rescues inhibition of lincRNAp21 on bladder cancer survival.
LncUCA1	Significantly expressed in bladder cancer tissues compared to normal tissue. Reduces ROS production, rescues mitochondrial function, upregulates glutaminase levels, and increases GLS1 and GLS2 mRNA expression. Interferes with miR16's tumor suppressor role in bladder cancer cells. Regulates redox state and glutamine metabolism contributing to tumorigenesis.
Tricarboxylic Acid (TCA) Cycle	
miR-181a, miR-183, let-7	Target isocitrate dehydrogenase (IDH) and PDK1 in the TCA cycle.
LncGAS5	Acts as a tumor suppressor by blocking TCA cycle regulation. Overexpression decreases cell viability through inhibition of enhancer of zest homolog 2 (EZH2) transcription by interacting with E2F4, resulting in increased expression of miR101.
Oxidative Phosphorylation	
miR-195	Targets glutamate dehydrogenase 1 (GLUD1) and ADPribosylation protein (ARL2) in bladder tumor cells. Suppresses proliferation, migration, invasion, and apoptosis in clear cell renal cell carcinoma cell line. In prostate cancer, inhibits cancer growth and epithelial-mesenchymal transition (EMT).
miR-17-3p	Inhibits antioxidant enzymes, manganese superoxide dismutase, glutathione peroxidase 2, and thioredoxin reductase 2 in prostate cancer cell lines, sensitizing them to ionizing radiation. Improves radiotherapy for aggressive tumors, including advanced prostate cancer.
circ_0004463, miR- 380-3p	circ_0004463 downregulated in bladder cancer tissue acts as a tumor suppressor. miR3803p upregulated in bladder cancer, promotes cell proliferation by mitochondrial metabolism.
Lipid Metabolism	
AnxA3	Regulates differentiation of adipose tissue into fat cells. Decreased expression of 36kDa AnxA3 and increased expression of 33kDa AnxA3 in renal cell carcinoma. Decreased expression is associated with low lipid storage in ccRCC cells.
miR-185, miR-342	Regulates lipid and cholesterol production by inhibiting sterol regulatory element binding proteins (SREBP)1 and 2. Downregulates fatty acid synthase (FASN) and 3hydroxy3methylglutaryl CoA reductase (HMGCR) in prostate cancer cell lines, inhibiting cell growth, migration, and invasion.
miR-101	Suppresses COX-2 expression, inhibiting cell and tumor growth in prostate cancer.

### 3.1 Glucose metabolism

Deregulated glucose metabolism is a defining characteristic of cancer (Ward and Thompson, 2012; Pavlova and Thompson, 2016). miRNAs can target glucose metabolic enzymes either directly or indirectly through intermediary loops, for instance, miR-34a/c and miR-374a directly target LDH mRNA in pancreatic can (Wang et al., 2015). Interestingly, miR-34a is decreased in bladder cancer. Transfection of miR-miR-34a mimics upregulated expression of PTEN, thereby decreasing cancer cell growth and viability (Hoque et al., 2003; Vinall et al., 2012). Similarly, miR-34a expression is inhibited in prostate cancer tissue (Duan et al., 2015). This evidence suggests the tumor-suppressor role of miR-34a in bladder and prostate cancer. On the contrary, miR-34a is upregulated in chromophobe renal cell carcinoma, where MET and E2F3 were significantly upregulated, while TP53INP2 and SOX2 are downregulated. Another miRNA, miR-155 targets C/enhancer-binding protein alpha which is a transcription factor for miR-143 that inhibits hexokinase 2 (Jiang et al., 2012). Cell-free miR-155 expression is correlated with the stage, and grade of bladder cancer and renal cell carcinoma (Aveta et al., 2023). Further, miR-124 regulates genes of both pyruvate kinase M2 (PKM2) and pentose phosphate pathway (PPP) in prostate cancer and bladder cancer (Sun et al., 2012; Qiu et al., 2015; Taniguchi et al., 2015). The miR-124 was found to be significantly lower in renal cell carcinoma tissue compared to the normal tissue. However, the involvement of miR-34a, miR374a, and miR-124 has not been extensively studied in the mitochondrial metabolism of genitourinary cancer.

LncUCA1 activates mTOR, by inducing signal transducer and activator of transcription 3 protein, and inhibiting miR-143, thereby upregulating hexokinase 2 and glycolysis in bladder cancer (Li et al., 2014). Another lncRNA PCGEM1 is shown to be overexpressed in prostate and renal cell carcinoma, suggesting its role as an oncogenic ncRNA. Interestingly, this promotes glucose uptake for aerobic glycolysis and couples it with PPP to facilitate nucleotide and lipid biosynthesis, thereby generating NADPH for redox homeostasis (Hung et al., 2014).

Hu et al. (2017) discovered that the lncCASC8 gene is reduced in high-grade bladder cancer. CASC8 protein binds to the fibroblast growth factor receptor 1 (FGFR1) and abrogates lactate dehydrogenase-A phosphorylation, thereby reducing glycolysis, and inhibiting bladder cancer cell growth. In RCC, the knockdown of the lncFILNC1 gene increases the c-Myc protein level by the FILNC1-AUF1-c-Myc signaling axis under glucose starvation conditions (Xiao et al., 2017). Another, lncRNA, SLC16A1-AS1 was shown to improve glycolysis and mitochondrial respiration by increasing ATP synthesis in bladder cancer. This leads to an increase in the proliferation of bladder cancer by fatty acid -oxidation (Logotheti et al., 2020).

During hypoxia, RCC cells show upregulated expression of miR-210. This study supports that miR-210 upregulation in RCC is predominantly mediated by hypoxia-inducible factor- 1 (Juan et al., 2010; McCormick et al., 2013; Wach et al., 2013). Another study has found that miR-429 decreased RCC cell growth and viability by inhibiting PDCD4, VEGF, c-myc, and AKT pathways (Su et al., 2020). miR-210 was found to be upregulated in the blood serum of bladder cancer patients, and its levels increase with the progression of the disease (Yang et al., 2017). Furthermore, miR-210-3p was shown to regulate bladder cancer growth, invasion, and metastasis by targeting FGFRL1. Similarly, in prostate cancer, overexpression of miR-210-3p was found significantly higher in tumor tissues. In addition, the expression levels of miR-210-3p are correlated with bone metastasis in prostate tissue (Ren et al., 2017).

### 3.2 Glutamine metabolism

**FIGURE 3:**
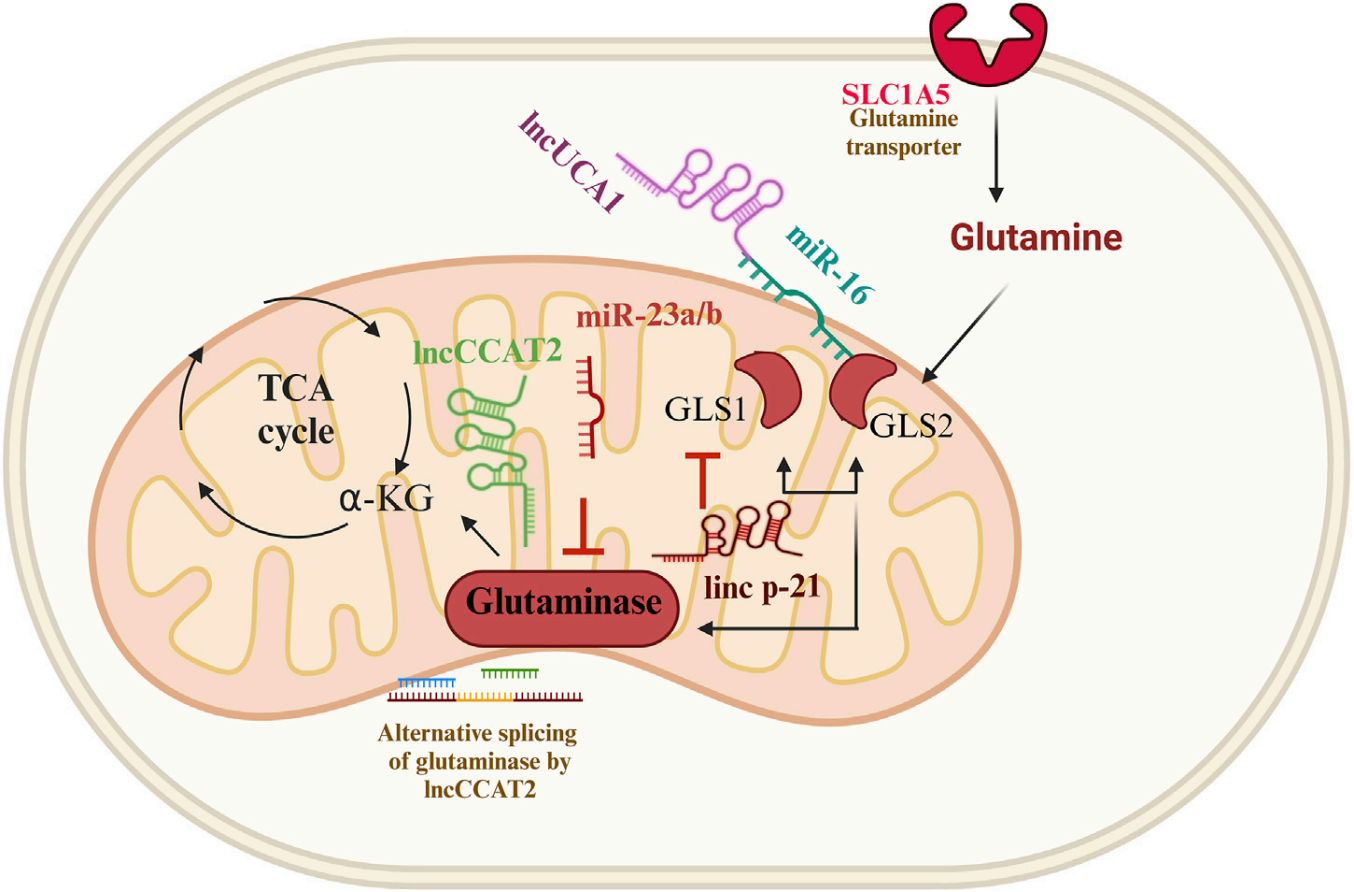
Glutamine metabolism and ncRNAs in genitourinary cancer. Four distinct paradigms of glutaminase (GLS) modulation have surfaced in genitourinary cancer. The first involves the inhibition of bladder cancer proliferation by the lncRNA-p21, which negatively regulates the expression of glutaminase, glutamate, and α-ketoglutarate; the second paradigm entails the simultaneous inhibition of GLS2; the third paradigm revolves around the allele-specific metabolic reprogramming of glutamine through the lncRNA CCAT2; the fourth paradigm involves the UCA1-miR-16-GLS2 axis, which regulates redox state and glutamine metabolism, contributing to tumorigenesis.

Glutamine is a key nutrient that fuels cellular metabolism, especially in cancer cells (Figure 3). Glutamine is transformed into glutamate through the action of an enzyme called glutaminase (GLS). There are two types of glutaminase, kidney type (GLS) and liver type (GLS2) (Katt et al., 2017). Two paradigms of GLS modulation have emerged: the first is the concurrent regulation by miR-23a/b and the lncRNA CCAT2, and the second is the allele-specific metabolic reprogramming of glutamine by CCAT2 (Redis et al., 2016). Additionally, miR-23b and miR-23b share the same transcript, with the latter inhibiting GLS translation. Importantly, miR-23b downregulates POX/PRODH in renal cell carcinoma. Findings from the MYC-inducible human Burkitt lymphoma model P493 and PC3 human prostate cancer cells affirm that MYC primarily suppresses POX/PRODH expression by up-regulating miR-23b (Liu et al., 2010; Liu et al., 2012).

Recent studies found that lincRNA-p21 can inhibit bladder cancer proliferation by negatively regulating glutaminase, glutamate, and α-ketoglutarate expression (Benitez et al., 2021; Scholda et al., 2023). Overexpression of glutaminase rescued inhibitory nature of lincRNA-p21 on bladder cancer survival. Additionally, the abundance of lincRNA-p21 and glutaminase dictates the response of bladder cancer cells to BPTES (glutaminase inhibitor) treatment. In bladder cancer tissues the lincRNA-p21 expression is significantly decreased, while glutaminase mRNA level is increased compared to normal tissues (Zhou et al., 2019). It has been observed that in prostate cancer, lincRNA-p21 downregulates and stimulates apoptosis. On the other hand, the malignant prostate tissues showed a reduction in the expression of the downstream genes of p53 (Wang et al., 2017). Moreover, lncRNA-p21 augments the methylation of STAT3 by enhancer of zeste homolog 2 (EZH2), leading to prostate cancer neuroendocrine transdifferentiation (Luo et al., 2019).

LncRNA UCA1 is a critical player in bladder cancer cells. LncUCA1 is significantly expressed in bladder cancer tissues compared to normal tissue. LncUCA1 was shown to reduce ROS production to rescue mitochondrial function by altering glutamine metabolism. LncUCA1 can also upregulate glutaminase levels and increase mRNA expression of both GLS1 and GLS2. miR-16 directly binds to the 3′UTR of GLS2 mRNA to inhibit bladder cancer growth, whereas lncUCA1 was found to interfere with miR-16’s tumor suppressor role in bladder cancer cells. This study indicates that the UCA1-miR-16-GLS2 axis regulates redox state, and glutamine metabolism, contributing to tumorigenesis (Li et al., 2015).

### 3.3 Tricarboxylic acid (TCA) Cycle

In addition to GLS, other key enzymes involved in TCA cycle are targeted by ncRNAs, such as isocitrate dehydrogenase (IDH) by miR-181a and miR-183, or PDK1 by let-7 (Fedele et al., 2022). LncGAS5 acts as a tumor suppressor by blocking TCA cycle regulation (Sang et al., 2021). Another study found that GAS5 overexpression decreased cell viability through inhibition of enhancer of zest homolog 2 (EZH2) transcription by interacting with E2F4, which resulted in increased expression of miR-101. Treatment with Gambogic acid elevated the level of GAS5 and its knockdown abolished gambogic acid-induced apoptosis in bladder cancer cells (Wang et al., 2018). However, overexpression of GAS5 can inhibit cell proliferation by inhibiting androgen receptor transactivation in castration-resistant prostate cancer cells (CRPC). Interestingly, a feedback loop has been discovered where suppressed androgen receptor downregulates the expression of GAS5, leading to increased transcription activity in CRPC. This study suggests that GAS5 plays a key role in androgen receptor axis activity and CRPC progression (Lv et al., 2021). GAS5 and miR-34a were positively correlated in renal cell carcinoma, however further studies are required to explore the effect of GAS5 on mitochondrial metabolism of renal cell cancer.

### 3.4 Oxidative phosphorylation

Oxidative phosphorylation uses the reduction of oxygen to produce high-energy ATP by the chemiosmotic electron transfer chain (ETC). In tumor cells, the functional electron transport chain is essential for promoting tumor growth by enabling the proliferation of cells through the mitochondrial complex I and III (Nolfi-Donegan et al., 2020; Ojha et al., 2022). A recent study used bioinformatics analysis to screen candidate target genes of miR-195 in bladder cancer, to identify which genes may play a role in regulating mitochondrial function. The analysis found that glutamate dehydrogenase 1 (GLUD1) and ADP- ADP-ribosylation protein (ARL2) were the ideal targets for miR-195 (Li et al., 2017). In bladder tumor cells, miR-195 directly inhibited ARL2 mRNA and protein levels, indicating that miR-195 may function as a tumor suppressor gene (Yu et al., 2018). LncUCA1 acts as a competing endogenous RNA to decrease the expression level of miR-195, resulting in increased ARL2 expression. This study highlights that the UCA1-miR-195-ARL2 signaling axis sustains mitochondrial metabolism in bladder cancer (Li et al., 2017; Newman et al., 2017).

Overexpression of miR-195 has been found to suppress the proliferation, migration, invasion, and apoptosis of a human clear cell renal cell carcinoma cell line, by inhibiting both the MAPK signaling pathways (Sun et al., 2016). Similarly, in prostate cancer, overexpression of miR-195 significantly inhibits cancer growth and epithelial-mesenchymal transition (EMT). This study further indicated that miR-195 inhibitor rescued the effect of 5-azacytidine on cell viability and metastatic potential of prostate cancer cells (Liu et al., 2015).

Conventional radiotherapy can adaptively induce antioxidant enzyme expression, manganese superoxide dismutase, glutathione peroxidase 2, and thioredoxin reductase 2, promoting therapeutic resistance (Liu et al., 2022). The overexpression of miR-17-3p, inhibits these three major antioxidant enzymes, thereby sensitizing prostate cancer cell lines to ionizing radiation. Therein, inhibition of NFκB-mediated protein activation has been shown to improve radiotherapy for aggressive tumors, including advanced prostate cancer (Xu et al., 2010; Xu et al., 2018). A new study has revealed that bladder cancer tissue samples and cells have significantly downregulated circ_0004463, indicating circ_0004463 role as a tumor suppressor. On the contrary, miR-380-3p was found to be upregulated in bladder cancer. It provides bladder cancer cell proliferation by mitochondrial metabolism, suggesting miR-308-3p role as a tumor promoter (Wu et al., 2020b).

### 3.5 Lipid metabolism

LncRNAs play a significant role in reprogramming of cancer lipid metabolism by regulating the expression of multiple signaling pathways during tumor development (Sellitto et al., 2021). LncRNA phospholipid-binding protein annexin A3 (AnxA3) negatively regulates the differentiation of adipose tissue into fat cells. There are two subtypes of AnxA3: 33 kDa and 36 kDa. The expression of 36 kDa AnxA3 is significantly decreased in renal cell carcinoma (ccRCC), while the expression of 33 kDa AnxA3 is increased, resulting overall decrease in AnxA3 expression. When ccRCC cells were exposed to an adipose culture medium, the expression of 36 kDa AnxA3 was found to be low, indicating that AnxA3 plays a negative role in the storage of lipids in ccRCC cells (Gu et al., 2009). Therefore, the impact of AnxA3 on RCC and its underlying mechanisms requires further investigation.

In prostate cancer cell lines, LNCaP and C4-2B, miR-185 and miR-342 regulate lipid and cholesterol production by inhibiting sterol regulatory element-binding proteins (SREBP)-1 and −2. This downregulates fatty acid synthase (FASN) and 3-hydroxy-3-methylglutaryl CoA reductase (HMGCR), inhibiting cell growth, migration, and invasion (Li et al., 2013). The miR-17/92 cluster targets peroxisome proliferator-activated receptor α (PPARA), a key lipid metabolism regulator. Testosterone and 1,25-dihydroxy vitamin D3 downregulate miR-17/92, relieving its inhibitory effect on PPARA, promoting lipid synthesis, and slowing tumor progression (Wang et al., 2013). Furthermore, miR-101 suppresses COX-2 expression, inhibiting cell and tumor growth in prostate cancer (Hao et al., 2011). These findings suggest that targeting abnormal lipid metabolism is a promising therapeutic approach for prostate cancer.

It has been shown by a recent study that certain metabolism-related lncRNA, such as LINC02004, DUXAP8, PWAR6, and AC073335, are abnormally regulated in bladder cancer (Zhang et al., 2019; Cui et al., 2021; Li et al., 2021; Wan et al., 2021; Wu et al., 2021). However, it is important to note that these lncRNA are not known to be involved in the regulation of lipid metabolism.

## 4 Therapeutic implications

Blocking dysfunctional metabolic pathways such as glucose, fatty acid, and amino acid oxidation represent promising therapeutic windows in cancer (Winkle et al., 2021; Clemente-Suárez et al., 2023). ncRNAs are potential candidates as they inhibit metabolic pathways by targeting multiple key genes. (Winkle et al., 2021). lncRNAs are very specific to their location and highly expressed in cancer. These characteristic features make them crucial candidates for cancer diagnosis and treatment. The most well-recognized ncRNA is PCA3, which is used as a diagnostic biomarker for the detection of prostate cancer at early stages (Opoku Mensah et al., 2022). Additionally, lncMALAT1 detection has been patented in prostate cancer diagnosis (CN104498495). ncRNAs are currently in clinical cancer trials specifically designed to target metabolic enzymes (Toden et al., 2021). LncUCA1 was found sensitive for bladder cancer, various clinical trials are underway to use it as a diagnostic marker in bladder cancer (Li et al., 2014; Li et al., 2015; Li et al., 2017; Ghafouri-Fard and Taheri, 2019). Largely, non-coding RNAs are key regulators in metabolism and major signaling pathways, which can be subjugated as therapeutic targets in the management of genitourinary cancer.

## 5 Future prospective

In recent years, the discovery of numerous mt-ncRNAs has advanced our knowledge of mitochondrial transcriptome and metabolism. Despite the relatively small size and limited protein-coding capacity of the mitochondrial genome, it possesses a unique profile of ncRNAs. While only a small subset has undergone thorough investigation, it has been established that mt-ncRNAs play pivotal roles in regulating mitochondrial gene expression and metabolism, among other functions. Although their applications are in early stages, with some undergoing clinical trials, due to their diverse roles in pathogenesis, mt-ncRNAs show promise as potential biomarkers, therapeutic targets, and even therapeutic RNA medications, particularly when it comes to genitourinary cancers. Recently, Next-Generation Sequencing Technology (NGS) revealed numerous ncRNAs as novel markers for diagnosing genitourinary cancers, including Renal Cell Carcinoma, Bladder Cancer, Prostate Cancer, Testicular, and Penile Cancers. Besides miRNAs and mRNAs being used for genitourinary cancer diagnosis, a significant presence of lncRNAs in human serum can be detected using unbiased high-throughput technologies such as genome tiling expression microarrays or deep-sequencing of serum samples via RNA-sequencing. Various therapeutic approaches targeting lncRNAs are currently under exploration. One direct strategy involves silencing the elevated levels of oncogenic lncRNAs through small interfering RNA. siRNAs designed to target specific lncRNAs have proven effective in reducing their expression in various genitourinary cancers. Additional potential agents for targeting lncRNAs include DNAzymes, single-stranded DNA molecules capable of cleaving complementary sequences, engineered based on naturally occurring RNA-based ribozymes. Advancements in fluorescent probe design, imaging technology, and image processing now allow precise identification of (sub)cellular localization and measurement of absolute expression levels of native ncRNA transcripts in individual cells with single-molecule precision *in situ*. This would provide a better understanding of the interaction between ncRNA and mitochondrial metabolism in genitourinary cancers. Understanding the molecular characteristics of lncRNAs and their roles in both healthy and cancerous cells could offer valuable insights into tumor biology, providing, previously unknown, potential therapeutic avenues for genitourinary cancers.

## 6 Conclusion

Metabolic reprogramming is a hallmark of cancer, which poses a major challenge for cancer management. Therefore, the molecular pathways responsible for the development of metabolic reprogramming need to be studied carefully to develop effective therapeutic strategies. In the last decades, ncRNAs have been shown as a novel cell function regulatory mechanism. Dysregulation of ncRNAs is shown to be involved in the cancer progression. At present, few miRNA therapies in cardiovascular disease are already undergoing clinical evaluation. For a few years, various new tumor-targeted ncRNAs-based cancer therapeutics have been an active area of investigation. Recent studies have shown that ncRNAs are vital factors in metabolic pathway regulation, and their potential as therapeutic targets is considerable for the management of cancer. However, more pre-clinical studies are needed to explore ncRNA potential in regulating metabolic reprogramming in genitourinary cancers. ncRNAs-based strategies can establish a prerequisite role in the diagnosis and therapy of genitourinary cancers.

## References

[B1] Arnaiz E. Miar A. Dias Junior A. G. Prasad N. Schulze U. Waithe D. (2021). Hypoxia regulates endogenous double-stranded RNA production via reduced mitochondrial DNA transcription. Front. Oncol. 11, 779739. 10.3389/fonc.2021.779739 34900733 PMC8651540

[B2] Aveta A. Cilio S. Contieri R. Spena G. Napolitano L. Manfredi C. (2023). Urinary MicroRNAs as biomarkers of urological cancers: a systematic review. Int. J. Mol. Sci. 24 (13), 10846. 10.3390/ijms241310846 37446024 PMC10341705

[B3] Benitez J. C. Campayo M. Díaz T. Ferrer C. Acosta-Plasencia M. Monzo M. (2021). Lincp21-RNA as predictive response marker for preoperative chemoradiotherapy in rectal cancer. J. Pers. Med. 11 (5), 420. 10.3390/jpm11050420 34065723 PMC8156811

[B4] Bismar T. A. Demichelis F. Riva A. Kim R. Varambally S. He L. (2006). Defining aggressive prostate cancer using a 12-gene model. Neoplasia 8 (1), 59–68. 10.1593/neo.05664 16533427 PMC1584291

[B5] Borgna V. Villegas J. Burzio V. A. Belmar S. Araya M. Jeldes E. (2017). Mitochondrial ASncmtRNA-1 and ASncmtRNA-2 as potent targets to inhibit tumor growth and metastasis in the RenCa murine renal adenocarcinoma model. Oncotarget 8 (27), 43692–43708. 10.18632/oncotarget.18460 28620146 PMC5546434

[B6] Borgna V. Lobos-González L. Guevara F. Landerer E. Bendek M. Ávila R. (2020). Targeting antisense mitochondrial noncoding RNAs induces bladder cancer cell death and inhibition of tumor growth through reduction of survival and invasion factors. J. Cancer 11 (7), 1780–1791. 10.7150/jca.38880 32194789 PMC7052861

[B7] Bosson A. D. Zamudio J. R. Sharp P. A. (2014). Endogenous miRNA and target concentrations determine susceptibility to potential ceRNA competition. Mol. Cell 56 (3), 347–359. 10.1016/j.molcel.2014.09.018 25449132 PMC5048918

[B8] Cantor J. R. Sabatini D. M. (2012). Cancer cell metabolism: one hallmark, many faces. Cancer Discov. 2 (10), 881–898. 10.1158/2159-8290.CD-12-0345 23009760 PMC3491070

[B9] Chen Y. B. Xu J. Skanderup A. J. Dong Y. Brannon A. R. Wang L. (2016). Molecular analysis of aggressive renal cell carcinoma with unclassified histology reveals distinct subsets. Nat. Commun. 7 (1), 13131. 10.1038/ncomms13131 27713405 PMC5059781

[B10] Chen L. Zhou M. Li H. Liu D. Liao P. Zong Y. (2023). Mitochondrial heterogeneity in diseases. Signal Transduct. Target Ther. 8 (1), 311. 10.1038/s41392-023-01546-w 37607925 PMC10444818

[B11] Cheng L. Montironi R. Davidson D. D. Lopez-Beltran A. (2009). Staging and reporting of urothelial carcinoma of the urinary bladder. Mod. Pathol. 22, S70–S95. 10.1038/modpathol.2009.1 19494855

[B12] Clemente-Suárez V. J. Martín-Rodríguez A. Yáñez-Sepúlveda R. Tornero-Aguilera J. F. (2023). Mitochondrial transfer as a novel therapeutic approach in disease diagnosis and treatment. IJMS 24 (10), 8848. 10.3390/ijms24108848 37240194 PMC10218908

[B13] Cui Y. Zhou Z. Chai Y. Che X. Zhang Y. (2021). Identification of a nomogram from ferroptosis-related long noncoding RNAs signature to analyze overall survival in patients with bladder cancer. J. Oncol. 2021, 1–18. 10.1155/2021/8533464 PMC8413054 34484338

[B14] Dasgupta S. Hoque M. O. Upadhyay S. Sidransky D. (2008). Mitochondrial Cytochrome B gene mutation promotes tumor growth in bladder cancer. Cancer Res. 68 (3), 700–706. 10.1158/0008-5472.CAN-07-5532 18245469

[B15] De Meerleer G. Khoo V. Escudier B. Joniau S. Bossi A. Ost P. (2014). Radiotherapy for renal-cell carcinoma. Lancet Oncol. 15 (4), e170–e177. 10.1016/S1470-2045(13)70569-2 24694640

[B16] DeBerardinis R. J. Chandel N. S. (2016). Fundamentals of cancer metabolism. Sci. Adv. 2 (5), e1600200. 10.1126/sciadv.1600200 27386546 PMC4928883

[B17] Delkov D. Yoanıdu L. Tomov D. Stoyanova R. Dechev I. Uzunova Y. (2022). Oncometabolites in urine - a new opportunity for detection and prognosis of the clinical progress of verified prostate cancer-a pilot study. Turk J. Med. Sci. 52 (3), 699–706. 10.55730/1300-0144.5363 36326306 PMC10390161

[B18] Dhir A. Dhir S. Borowski L. S. Jimenez L. Teitell M. Rötig A. (2018). Mitochondrial double-stranded RNA triggers antiviral signalling in humans. Nature 560 (7717), 238–242. 10.1038/s41586-018-0363-0 30046113 PMC6570621

[B19] Dong Y. Yoshitomi T. Hu J. F. Cui J. (2017). Long noncoding RNAs coordinate functions between mitochondria and the nucleus. Epigenetics Chromatin 10 (1), 41. 10.1186/s13072-017-0149-x 28835257 PMC5569521

[B20] Duan K. Ge Y. C. Zhang X. P. Wu S. Y. Feng J. S. Chen S. L. (2015). miR-34a inhibits cell proliferation in prostate cancer by downregulation of SIRT1 expression. Oncol. Lett. 10 (5), 3223–3227. 10.3892/ol.2015.3645 26722316 PMC4665267

[B21] Fedele M. Sgarra R. Battista S. Cerchia L. Manfioletti G. (2022). The epithelial-mesenchymal transition at the crossroads between metabolism and tumor progression. Int. J. Mol. Sci. 23 (2), 800. 10.3390/ijms23020800 35054987 PMC8776206

[B22] Gabriel K. Egan B. Lithgow T. (2003). Tom40, the import channel of the mitochondrial outer membrane, plays an active role in sorting imported proteins. EMBO J. 22 (10), 2380–2386. 10.1093/emboj/cdg229 12743032 PMC155987

[B23] Gallo Cantafio M. E. Torcasio R. Viglietto G. Amodio N. (2023). Non-coding RNA-dependent regulation of mitochondrial dynamics in cancer pathophysiology. ncRNA 9 (1), 16. 10.3390/ncrna9010016 36827549 PMC9964195

[B24] Gao S. Tian X. Chang H. Sun Y. Wu Z. Cheng Z. (2018). Two novel lncRNAs discovered in human mitochondrial DNA using PacBio full-length transcriptome data. Mitochondrion 38, 41–47. 10.1016/j.mito.2017.08.002 28802668

[B25] Ghafouri-Fard S. Taheri M. (2019). UCA1 long non-coding RNA: an update on its roles in malignant behavior of cancers. Biomed. Pharmacother. 120, 109459. 10.1016/j.biopha.2019.109459 31585301

[B26] Grillone K. Riillo C. Scionti F. Rocca R. Tradigo G. Guzzi P. H. (2020). Non-coding RNAs in cancer: platforms and strategies for investigating the genomic “dark matter.”. J. Exp. Clin. Cancer Res. 39 (1), 117. 10.1186/s13046-020-01622-x 32563270 PMC7305591

[B27] Gu S. Jin L. Zhang F. Sarnow P. Kay M. A. (2009). Biological basis for restriction of microRNA targets to the 3’ untranslated region in mammalian mRNAs. Nat. Struct. Mol. Biol. 16 (2), 144–150. 10.1038/nsmb.1552 19182800 PMC2713750

[B28] Hao Y. Gu X. Zhao Y. Greene S. Sha W. Smoot D. T. (2011). Enforced expression of miR-101 inhibits prostate cancer cell growth by modulating the COX-2 pathway *in vivo* . Cancer Prev. Res. 4 (7), 1073–1083. 10.1158/1940-6207.CAPR-10-0333 PMC3305792 21430074

[B29] Hoque M. O. Lee C. C. R. Cairns P. Schoenberg M. Sidransky D. (2003). Genome-wide genetic characterization of bladder cancer: a comparison of high-density single-nucleotide polymorphism arrays and PCR-based microsatellite analysis. Cancer Res. 63 (9), 2216–2222. 12727842

[B30] Hu R. Dunn T. A. Wei S. Isharwal S. Veltri R. W. Humphreys E. (2009). Ligand-independent androgen receptor variants derived from splicing of cryptic exons signify hormone-refractory prostate cancer. Cancer Res. 69 (1), 16–22. 10.1158/0008-5472.CAN-08-2764 19117982 PMC2614301

[B31] Hu R. Zhong P. Xiong L. Duan L. (2017). *Long noncoding RNA cancer susceptibility candidate 8* suppresses the proliferation of bladder cancer cells via regulating glycolysis. DNA Cell Biol. 36 (9), 767–774. 10.1089/dna.2017.3785 28759252

[B32] Huang C. C. Liu H. Y. Hsu T. W. Lee W. C. (2022). Updates on the pivotal roles of mitochondria in urothelial carcinoma. Biomedicines 10 (10), 2453. 10.3390/biomedicines10102453 36289714 PMC9599371

[B33] Hung C. L. Wang L. Y. Yu Y. L. Chen H. W. Srivastava S. Petrovics G. (2014). A long noncoding RNA connects c-Myc to tumor metabolism. Proc. Natl. Acad. Sci. U. S. A. 111 (52), 18697–18702. 10.1073/pnas.1415669112 25512540 PMC4284533

[B34] Hyttinen J. M. T. Blasiak J. Kaarniranta K. (2023). Non-coding RNAs regulating mitochondrial functions and the oxidative stress response as putative targets against age-related macular degeneration (AMD). IJMS 24 (3), 2636. 10.3390/ijms24032636 36768958 PMC9917342

[B35] Jiang S. Zhang L. F. Zhang H. W. Hu S. Lu M. H. Liang S. (2012). A novel miR-155/miR-143 cascade controls glycolysis by regulating hexokinase 2 in breast cancer cells. EMBO J. 31 (8), 1985–1998. 10.1038/emboj.2012.45 22354042 PMC3343331

[B36] Juan D. Alexe G. Antes T. Liu H. Madabhushi A. Delisi C. (2010). Identification of a microRNA panel for clear-cell kidney cancer. Urology 75 (4), 835–841. 10.1016/j.urology.2009.10.033 20035975

[B37] Katt W. P. Lukey M. J. Cerione R. A. (2017). A tale of two glutaminases: homologous enzymes with distinct roles in tumorigenesis. Future Med. Chem. 9 (2), 223–243. 10.4155/fmc-2016-0190 28111979 PMC5558546

[B38] Kaur B. Sohrabi Y. Achreja A. Lisanti M. P. Martinez-Outschoorn U. E. (2023). Editorial: hallmark of cancer: reprogramming of cellular metabolism. Front. Oncol. 11 (12), 1126913. 10.3389/fonc.2022.1126913 PMC9874241 36713555

[B39] Konety B. R. Joslyn S. A. (2003). Factors influencing aggressive therapy for bladder cancer: an analysis of data from the SEER program. J. Urology 170 (5), 1765–1771. 10.1097/01.ju.0000091620.86778.2e 14532772

[B40] Lavallee E. Sfakianos J. P. Mulholland D. J. (2021). Tumor heterogeneity and consequences for bladder cancer treatment. Cancers (Basel) 13 (21), 5297. 10.3390/cancers13215297 34771460 PMC8582570

[B41] Li X. Chen Y. T. Josson S. Mukhopadhyay N. K. Kim J. Freeman M. R. (2013). MicroRNA-185 and 342 inhibit tumorigenicity and induce apoptosis through blockade of the SREBP metabolic pathway in prostate cancer cells. PLoS ONE 8 (8), e70987. Campbell M. 10.1371/journal.pone.0070987 23951060 PMC3739799

[B42] Li Z. Li X. Wu S. Xue M. Chen W. (2014). Long non-coding RNA UCA1 promotes glycolysis by upregulating hexokinase 2 through the mTOR-STAT3/microRNA143 pathway. Cancer Sci. 105 (8), 951–955. 10.1111/cas.12461 24890811 PMC4317864

[B43] Li H. J. Li X. Pang H. Pan J. J. Xie X. J. Chen W. (2015). Long non-coding RNA UCA1 promotes glutamine metabolism by targeting miR-16 in human bladder cancer. Jpn. J. Clin. Oncol. 45 (11), 1055–1063. 10.1093/jjco/hyv132 26373319

[B44] Li H. J. Sun X. M. Li Z. K. Yin Q. W. Pang H. Pan J. J. (2017). LncRNA UCA1 promotes mitochondrial function of bladder cancer via the MiR-195/ARL2 signaling pathway. Cell Physiol. Biochem. 43 (6), 2548–2561. 10.1159/000484507 29130995

[B45] Li W. Xu C. Guo J. Liu K. Hu Y. Wu D. (2020). Cis- and trans-acting expression quantitative trait loci of long non-coding RNA in 2,549 cancers with potential clinical and therapeutic implications. Front. Oncol. 10, 602104. 10.3389/fonc.2020.602104 33194770 PMC7604522

[B46] Li M. Yang B. Li X. Ren H. Zhang L. Li L. (2021). Identification of prognostic factors related to super enhancer-regulated ceRNA network in metastatic lung adenocarcinoma. IJGM 14, 6261–6275. 10.2147/IJGM.S332317 PMC8493278 34629892

[B47] Liang H. Liu J. Su S. Zhao Q. (2021). Mitochondrial noncoding RNAs: new wine in an old bottle. RNA Biol. 18 (12), 2168–2182. 10.1080/15476286.2021.1935572 34110970 PMC8632133

[B48] Linehan W. M. Ricketts C. J. (2019). The Cancer Genome Atlas of renal cell carcinoma: findings and clinical implications. Nat. Rev. Urol. 16 (9), 539–552. 10.1038/s41585-019-0211-5 31278395

[B49] Liu X. Shan G. (2021). Mitochondria encoded non-coding RNAs in cell physiology. Front. Cell Dev. Biol. 9, 713729. 10.3389/fcell.2021.713729 34395442 PMC8362354

[B50] Liu W. Zabirnyk O. Wang H. Shiao Y. H. Nickerson M. L. Khalil S. (2010). miR-23b targets proline oxidase, a novel tumor suppressor protein in renal cancer. Oncogene 29 (35), 4914–4924. 10.1038/onc.2010.237 20562915 PMC4398970

[B51] Liu W. Le A. Hancock C. Lane A. N. Dang C. V. Fan T. W. M. (2012). Reprogramming of proline and glutamine metabolism contributes to the proliferative and metabolic responses regulated by oncogenic transcription factor c-MYC. Proc. Natl. Acad. Sci. U. S. A. 109 (23), 8983–8988. 10.1073/pnas.1203244109 22615405 PMC3384197

[B52] Liu C. Guan H. Wang Y. Chen M. Xu B. Zhang L. (2015). miR-195 inhibits EMT by targeting FGF2 in prostate cancer cells. PLoS One 10 (12), e0144073. 10.1007/s11427-020-1631-9 26650737 PMC4674136

[B53] Liu X. Wang X. Li J. Hu S. Deng Y. Yin H. (2019). The identification of mecciRNAs and their roles in mitochondrial entry of proteins. Mol. Biol. 10.1101/668665 32048164

[B54] Liu X. Wang X. Li J. Hu S. Deng Y. Yin H. (2020). Identification of mecciRNAs and their roles in the mitochondrial entry of proteins. Sci. China Life Sci. 63 (10), 1429–1449. 10.1007/s11427-020-1631-9 32048164

[B55] Liu R. Bian Y. Liu L. Liu L. Liu X. Ma S. (2022). Molecular pathways associated with oxidative stress and their potential applications in radiotherapy (Review). Int. J. Mol. Med. 49 (5), 65. 10.3892/ijmm.2022.5121 35293589 PMC8989428

[B56] Logotheti S. Marquardt S. Gupta S. K. Richter C. Edelhäuser B. A. H. Engelmann D. (2020). LncRNA-SLC16A1-AS1 induces metabolic reprogramming during Bladder Cancer progression as target and co-activator of E2F1. Theranostics 10 (21), 9620–9643. 10.7150/thno.44176 32863950 PMC7449907

[B57] Luo J. Wang K. Yeh S. Sun Y. Liang L. Xiao Y. (2019). LncRNA-p21 alters the antiandrogen enzalutamide-induced prostate cancer neuroendocrine differentiation via modulating the EZH2/STAT3 signaling. Nat. Commun. 10 (1), 2571. 10.1038/s41467-019-09784-9 31189930 PMC6561926

[B58] Lv S. Pu X. Luo M. Wen H. Xu Z. Wei Q. (2021). Long noncoding RNA GAS5 interacts and suppresses androgen receptor activity in prostate cancer cells. Prostate 81 (12), 893–901. 10.1002/pros.24186 34184786

[B59] Ma L. Bajic V. B. Zhang Z. (2013). On the classification of long non-coding RNAs. RNA Biol. 10 (6), 925–933. 10.4161/rna.24604 23696037 PMC4111732

[B60] Mattick J. S. Makunin I. V. (2006). Non-coding RNA. Hum. Mol. Genet. 15 (1), R17–R29. 10.1093/hmg/ddl046 16651366

[B61] Mattick J. S. Amaral P. P. Carninci P. Carpenter S. Chang H. Y. Chen L. L. (2023). Long non-coding RNAs: definitions, functions, challenges and recommendations. Nat. Rev. Mol. Cell Biol. 24 (6), 430–447. 10.1038/s41580-022-00566-8 36596869 PMC10213152

[B62] McCormick R. I. Blick C. Ragoussis J. Schoedel J. Mole D. R. Young A. C. (2013). miR-210 is a target of hypoxia-inducible factors 1 and 2 in renal cancer, regulates ISCU and correlates with good prognosis. Br. J. Cancer 108 (5), 1133–1142. 10.1038/bjc.2013.56 23449350 PMC3619073

[B63] Mercer T. R. Neph S. Dinger M. E. Crawford J. Smith M. A. Shearwood A. M. J. (2011). The human mitochondrial transcriptome. Cell 146 (4), 645–658. 10.1016/j.cell.2011.06.051 21854988 PMC3160626

[B64] Newman L. E. Schiavon C. R. Zhou C. Kahn R. A. (2017). The abundance of the ARL2 GTPase and its GAP, ELMOD2, at mitochondria are modulated by the fusogenic activity of mitofusins and stressors. PLoS One 12 (4), e0175164. 10.1371/journal.pone.0175164 28380071 PMC5381910

[B65] Nolfi-Donegan D. Braganza A. Shiva S. (2020). Mitochondrial electron transport chain: oxidative phosphorylation, oxidant production, and methods of measurement. Redox Biol. 37, 101674. 10.1016/j.redox.2020.101674 32811789 PMC7767752

[B66] O’Brien J. Hayder H. Zayed Y. Peng C. (2018). Overview of MicroRNA biogenesis, mechanisms of actions, and circulation. Front. Endocrinol. (Lausanne). 9, 402. 10.3389/fendo.2018.00402 30123182 PMC6085463

[B67] Ojha R. Tantray I. Rimal S. Mitra S. Cheshier S. Lu B. (2022). Regulation of reverse electron transfer at mitochondrial complex I by unconventional Notch action in cancer stem cells. Dev. Cell 57 (2), 260–276.e9. 10.1016/j.devcel.2021.12.020 35077680 PMC8852348

[B68] Olgun G. Sahin O. Tastan O. (2018). Discovering lncRNA mediated sponge interactions in breast cancer molecular subtypes. BMC Genomics 19 (1), 650. 10.1186/s12864-018-5006-1 30180792 PMC6122485

[B69] Opoku Mensah B. Fondjo L. A. Owiredu WKBA Adusei B. (2022). Urinary PCA3 a superior diagnostic biomarker for prostate cancer among Ghanaian men. Dis. Markers 2022, 1686991. 10.1155/2022/1686991 36246565 PMC9568348

[B70] Pavlova N. N. Thompson C. B. (2016). The emerging hallmarks of cancer metabolism. Cell Metab. 23 (1), 27–47. 10.1016/j.cmet.2015.12.006 26771115 PMC4715268

[B71] Peng Y. Croce C. M. (2016). The role of MicroRNAs in human cancer. Sig Transduct. Target Ther. 1 (1), 15004. 10.1038/sigtrans.2015.4 PMC5661652 29263891

[B72] Pozzi A. Dowling D. K. (2019). The genomic origins of small mitochondrial RNAs: are they transcribed by the mitochondrial DNA or by mitochondrial pseudogenes within the nucleus (NUMTs)? Genome Biol. Evol. 11 (7), 1883–1896. 10.1093/gbe/evz132 31218347 PMC6619488

[B73] Qiu Z. Guo W. Wang Q. Chen Z. Huang S. Zhao F. (2015). MicroRNA-124 reduces the pentose phosphate pathway and proliferation by targeting PRPS1 and RPIA mRNAs in human colorectal cancer cells. Gastroenterology 149 (6), 1587–1598. 10.1053/j.gastro.2015.07.050 26248089

[B74] Rackham O. Shearwood A. M. J. Mercer T. R. Davies S. M. K. Mattick J. S. Filipovska A. (2011). Long noncoding RNAs are generated from the mitochondrial genome and regulated by nuclear-encoded proteins. RNA 17 (12), 2085–2093. 10.1261/rna.029405.111 22028365 PMC3222122

[B75] Ransohoff J. D. Wei Y. Khavari P. A. (2018). The functions and unique features of long intergenic non-coding RNA. Nat. Rev. Mol. Cell Biol. 19 (3), 143–157. 10.1038/nrm.2017.104 29138516 PMC5889127

[B76] Raza S. H. A. Wijayanti D. Pant S. D. Abdelnour S. A. Hashem N. M. Amin A. (2022). Exploring the physiological roles of circular RNAs in livestock animals. Res. Vet. Sci. 20 (152), 726–735. 10.1016/j.rvsc.2022.09.036 36270182

[B77] Redis R. S. Vela L. E. Lu W. Ferreira de Oliveira J. Ivan C. Rodriguez-Aguayo C. (2016). Allele-specific reprogramming of cancer metabolism by the long non-coding RNA CCAT2. Mol. Cell 61 (4), 640. 10.1016/j.molcel.2016.02.006 28934601

[B78] Ren D. Yang Q. Dai Y. Guo W. Du H. Song L. (2017). Oncogenic miR-210-3p promotes prostate cancer cell EMT and bone metastasis via NF-κB signaling pathway. Mol. Cancer 16 (1), 117. 10.1186/s12943-017-0688-6 28693582 PMC5504657

[B79] Ren B. Guan M. X. Zhou T. Cai X. Shan G. (2023). Emerging functions of mitochondria-encoded noncoding RNAs. Trends Genet. 39 (2), 125–139. 10.1016/j.tig.2022.08.004 36137834

[B80] Riscal R. Bull C. J. Mesaros C. Finan J. M. Carens M. Ho E. S. (2021). Cholesterol auxotrophy as a targetable vulnerability in clear cell renal cell carcinoma. Cancer Discov. 11 (12), 3106–3125. 10.1158/2159-8290.CD-21-0211 34244212 PMC8741905

[B81] Ross K. Jones R. J. (2017). Immune checkpoint inhibitors in renal cell carcinoma. Clin. Sci. (Lond) 131 (21), 2627–2642. 10.1042/CS20160894 29079639 PMC5869245

[B82] Sang L. Ju H. Q. Yang Z. Ge Q. Zhang Z. Liu F. (2021). Mitochondrial long non-coding RNA GAS5 tunes TCA metabolism in response to nutrient stress. Nat. Metab. 3 (1), 90–106. 10.1038/s42255-020-00325-z 33398195

[B83] Scheid A. D. Beadnell T. C. Welch D. R. (2021). Roles of mitochondria in the hallmarks of metastasis. Br. J. Cancer 124 (1), 124–135. 10.1038/s41416-020-01125-8 33144695 PMC7782743

[B84] Scholda J. Nguyen T. T. A. Kopp F. (2023). Long noncoding RNAs as versatile molecular regulators of cellular stress response and homeostasis. Hum. Genet., 02604-7. 10.1007/s00439-023-02604-7 PMC11294412 37782337

[B85] Sellitto A. Pecoraro G. Giurato G. Nassa G. Rizzo F. Saggese P. (2021). Regulation of metabolic reprogramming by long non-coding RNAs in cancer. Cancers (Basel) 13 (14), 3485. 10.3390/cancers13143485 34298698 PMC8308086

[B86] Shi Y. Downes M. Xie W. Kao H. Y. Ordentlich P. Tsai C. C. (2001). Sharp, an inducible cofactor that integrates nuclear receptor repression and activation. Genes Dev. 15 (9), 1140–1151. 10.1101/gad.871201 11331609 PMC312688

[B87] Shim E. H. Livi C. B. Rakheja D. Tan J. Benson D. Parekh V. (2014). L-2-Hydroxyglutarate: an epigenetic modifier and putative oncometabolite in renal cancer. Cancer Discov. 4 (11), 1290–1298. 10.1158/2159-8290.CD-13-0696 25182153 PMC4286872

[B88] Slack F. J. Chinnaiyan A. M. (2019). The role of non-coding RNAs in oncology. Cell 179 (5), 1033–1055. 10.1016/j.cell.2019.10.017 31730848 PMC7347159

[B89] Su Z. Jiang G. Chen J. Liu X. Zhao H. Fang Z. (2020). MicroRNA-429 inhibits cancer cell proliferation and migration by targeting AKT1 in renal cell carcinoma. Mol. Clin. Oncol. 12 (1), 75–80. 10.3892/mco.2019.1940 31814979 PMC6888107

[B90] Sullivan L. B. Martinez-Garcia E. Nguyen H. Mullen A. R. Dufour E. Sudarshan S. (2013). The proto-oncometabolite fumarate binds glutathione to amplify ROS-dependent signaling. Mol. Cell 51 (2), 236–248. 10.1016/j.molcel.2013.05.003 23747014 PMC3775267

[B91] Sun Y. Zhao X. Zhou Y. Hu Y. (2012). miR-124, miR-137 and miR-340 regulate colorectal cancer growth via inhibition of the Warburg effect. Oncol. Rep. 28 (4), 1346–1352. 10.3892/or.2012.1958 22895557

[B92] Sun P. Wang L. Lu Y. Liu Y. Li L. Yin L. (2016). MicroRNA-195 targets VEGFR2 and has a tumor suppressive role in ACHN cells via PI3K/Akt and Raf/MEK/ERK signaling pathways. Int. J. Oncol. 49 (3), 1155–1163. 10.3892/ijo.2016.3608 27572273

[B93] Sung H. Ferlay J. Siegel R. L. Laversanne M. Soerjomataram I. Jemal A. (2021). Global cancer statistics 2020: GLOBOCAN estimates of incidence and mortality worldwide for 36 cancers in 185 countries. CA Cancer J. Clin. 71 (3), 209–249. 10.3322/caac.21660 33538338

[B94] Taniguchi K. Ito Y. Sugito N. Kumazaki M. Shinohara H. Yamada N. (2015). Organ-specific PTB1-associated microRNAs determine expression of pyruvate kinase isoforms. Sci. Rep. 5 (1), 8647. 10.1038/srep08647 25721733 PMC4342556

[B95] Tantray I. Ojha R. Sharma A. P. (2023). Non-coding RNA and autophagy: finding novel ways to improve the diagnostic management of bladder cancer. Front. Genet. 13, 1051762. 10.3389/fgene.2022.1051762 36685879 PMC9845264

[B96] Toden S. Zumwalt T. J. Goel A. (2021). Non-coding RNAs and potential therapeutic targeting in cancer. Biochim. Biophys. Acta Rev. Cancer 1875 (1), 188491. 10.1016/j.bbcan.2020.188491 33316377 PMC7856203

[B97] Vartak R. Deng J. Fang H. Bai Y. (2015). Redefining the roles of mitochondrial DNA-encoded subunits in respiratory Complex I assembly. Biochim. Biophys. Acta 1852 (7), 1531–1539. 10.1016/j.bbadis.2015.04.008 25887158 PMC4433823

[B98] Vikramdeo K. S. Sharma A. Anand S. Sudan S. K. Singh S. Singh A. P. (2023). Mitochondrial alterations in prostate cancer: roles in pathobiology and racial disparities. Int. J. Mol. Sci. 24 (5), 4482. 10.3390/ijms24054482 36901912 PMC10003184

[B99] Villegas J. Burzio V. Villota C. Landerer E. Martinez R. Santander M. (2007). Expression of a no*vel non*-coding mitochondrial RNA in human proliferating cells. Nucleic Acids Res. 35 (21), 7336–7347. 10.1093/nar/gkm863 17962305 PMC2175360

[B100] Vinall R. L. Ripoll A. Z. Wang S. Pan C. deVere White R. W. (2012). MiR‐34a chemosensitizes bladder cancer cells to cisplatin treatment regardless of p53‐Rb pathway status. Intl J. Cancer 130 (11), 2526–2538. 10.1002/ijc.26256 PMC4568996 21702042

[B101] Wach S. Nolte E. Theil A. Stöhr C. T Rau T. Hartmann A. (2013). MicroRNA profiles classify papillary renal cell carcinoma subtypes. Br. J. Cancer 109 (3), 714–722. 10.1038/bjc.2013.313 23799849 PMC3738121

[B102] Wan J. Guo C. Fang H. Xu Z. Hu Y. Luo Y. (2021). Autophagy-related long non-coding RNA is a prognostic indicator for bladder cancer. Front. Oncol. 11, 647236. 10.3389/fonc.2021.647236 33869042 PMC8049181

[B103] Wang Y. Patti G. J. (2023). The Warburg effect: a signature of mitochondrial overload. Trends Cell Biol. 33 (12), 1014–1020. 10.1016/j.tcb.2023.03.013 37117116 PMC10600323

[B104] Wang G. Chen H. W. Oktay Y. Zhang J. Allen E. L. Smith G. M. (2010). PNPASE regulates RNA import into mitochondria. Cell 142 (3), 456–467. 10.1016/j.cell.2010.06.035 20691904 PMC2921675

[B105] Wang G. Shimada E. Koehler C. M. Teitell M. A. (2012). PNPASE and RNA trafficking into mitochondria. Biochim. Biophys. Acta 1819 (9–10), 998–1007. 10.1016/j.bbagrm.2011.10.001 22023881 PMC3267854

[B106] Wang W. L. W. Welsh J. Tenniswood M. (2013). 1,25-Dihydroxyvitamin D3 modulates lipid metabolism in prostate cancer cells through miRNA mediated regulation of PPARA. J. Steroid Biochem. Mol. Biol. 136, 247–251. 10.1016/j.jsbmb.2012.09.033 23059473

[B107] Wang J. Wang H. Liu A. Fang C. Hao J. Wang Z. (2015). Lactate dehydrogenase A negatively regulated by miRNAs promotes aerobic glycolysis and is increased in colorectal cancer. Oncotarget 6 (23), 19456–19468. 10.18632/oncotarget.3318 26062441 PMC4637298

[B108] Wang X. Ruan Y. Wang X. Zhao W. Jiang Q. Jiang C. (2017). Long intragenic non-coding RNA lincRNA-p21 suppresses development of human prostate cancer. Cell Prolif. 50 (2), e12318. 10.1111/cpr.12318 27976428 PMC6529152

[B109] Wang M. Guo C. Wang L. Luo G. Huang C. Li Y. (2018). Long noncoding RNA GAS5 promotes bladder cancer cells apoptosis through inhibiting EZH2 transcription. Cell Death Dis. 9 (2), 238. 10.1038/s41419-018-0264-z 29445179 PMC5833416

[B110] Ward P. S. Thompson C. B. (2012). Metabolic reprogramming: a cancer hallmark even warburg did not anticipate. Cancer Cell 21 (3), 297–308. 10.1016/j.ccr.2012.02.014 22439925 PMC3311998

[B111] Winkle M. El-Daly S. M. Fabbri M. Calin G. A. (2021). Noncoding RNA therapeutics — challenges and potential solutions. Nat. Rev. Drug Discov. 20 (8), 629–651. 10.1038/s41573-021-00219-z 34145432 PMC8212082

[B112] Wu Z. Sun H. Wang C. Liu W. Liu M. Zhu Y. (2020a). Mitochondrial genome-derived circRNA mc-COX2 functions as an oncogene in chronic lymphocytic leukemia. Mol. Ther. Nucleic Acids 20, 801–811. 10.1016/j.omtn.2020.04.017 32438315 PMC7240210

[B113] Wu S. Deng H. He H. Xu R. Wang Y. Zhu X. (2020b). The circ_0004463/miR-380-3p/FOXO1 axis modulates mitochondrial respiration and bladder cancer cell apoptosis. Cell Cycle 19 (24), 3563–3580. 10.1080/15384101.2020.1852746 33283616 PMC7781606

[B114] Wu J. Cai Y. Zhao G. Li M. (2021). A ten N6‐methyladenosine‐related long non‐coding RNAs signature predicts prognosis of triple‐negative breast cancer. Clin. Lab. Anal. 35 (6), e23779. 10.1002/jcla.23779 PMC8183938 33934391

[B115] Xiao Z. D. Han L. Lee H. Zhuang L. Zhang Y. Baddour J. (2017). Energy stress-induced lncRNA FILNC1 represses c-Myc-mediated energy metabolism and inhibits renal tumor development. Nat. Commun. 8 (1), 783. 10.1038/s41467-017-00902-z 28978906 PMC5627275

[B116] Xu Y. Fang F. Zhang J. Josson S. St Clair W. H. St Clair D. K. (2010). miR-17* suppresses tumorigenicity of prostate cancer by inhibiting mitochondrial antioxidant enzymes. PLoS One 5 (12), e14356. 10.1371/journal.pone.0014356 21203553 PMC3008681

[B117] Xu Z. Zhang Y. Ding J. Hu W. Tan C. Wang M. (2018). miR-17-3p downregulates mitochondrial antioxidant enzymes and enhances the radiosensitivity of prostate cancer cells. Mol. Ther. Nucleic Acids 13, 64–77. 10.1016/j.omtn.2018.08.009 30240971 PMC6143750

[B118] Xu Y. Qiu M. Shen M. Dong S. Ye G. Shi X. (2021). The emerging regulatory roles of long non-coding RNAs implicated in cancer metabolism. Mol. Ther. 29 (7), 2209–2218. 10.1016/j.ymthe.2021.03.017 33775912 PMC8261164

[B119] Yang X. Shi L. Yi C. Yang Y. Chang L. Song D. (2017). MiR-210-3p inhibits the tumor growth and metastasis of bladder cancer via targeting fibroblast growth factor receptor-like 1. Am. J. Cancer Res. 7 (8), 1738–1753. 28861329 PMC5574945

[B120] Yong C. Stewart G. D. Frezza C. (2020). Oncometabolites in renal cancer. Nat. Rev. Nephrol. 16 (3), 156–172. 10.1038/s41581-019-0210-z 31636445 PMC7030949

[B121] You M. Xie Z. Zhang N. Zhang Y. Xiao D. Liu S. (2023). Signaling pathways in cancer metabolism: mechanisms and therapeutic targets. Sig Transduct. Target Ther. 8 (1), 196. 10.1038/s41392-023-01442-3 PMC10172373 37164974

[B122] Yu W. Liang X. Li X. Zhang Y. Sun Z. Liu Y. (2018). MicroRNA-195: a review of its role in cancers. Onco Targets Ther. 11, 7109–7123. 10.2147/OTT.S183600 30410367 PMC6200091

[B123] Zampetaki A. Albrecht A. Steinhofel K. (2018). Long non-coding RNA structure and function: is there a link? Front. Physiol. 9, 1201. 10.3389/fphys.2018.01201 30197605 PMC6117379

[B124] Zarrabi K. Paroya A. Wu S. (2019). Emerging therapeutic agents for genitourinary cancers. J. Hematol. Oncol. 12 (1), 89. 10.1186/s13045-019-0780-z 31484560 PMC6727406

[B125] Zhang X. Li T. Wang J. Li J. Chen L. Liu C. (2019). Identification of cancer-related long non-coding RNAs using XGBoost with high accuracy. Front. Genet. 10, 735. 10.3389/fgene.2019.00735 31456817 PMC6701491

[B126] Zhao Q. Liu J. Deng H. Ma R. Liao J. Y. Liang H. (2020). Targeting mitochondria-located circRNA SCAR alleviates NASH via reducing mROS output. Cell 183 (1), 76–93. 10.1016/j.cell.2020.08.009 32931733

[B127] Zhou Q. Zhan H. Lin F. Liu Y. Yang K. Gao Q. (2019). LincRNA-p21 suppresses glutamine catabolism and bladder cancer cell growth through inhibiting glutaminase expression. Biosci. Rep. 39 (4), BSR20182372. 10.1042/BSR20182372 30902882 PMC6465205

[B128] Zhou W. Y. Cai Z. R. Liu J. Wang D. S. Ju H. Q. Xu R. H. (2020). Circular RNA: metabolism, functions and interactions with proteins. Mol. Cancer 19 (1), 172. 10.1186/s12943-020-01286-3 33317550 PMC7734744

[B129] Zhou S. Yu Q. Zhang L. Jiang Z. (2023). Cyclophilin D-mediated mitochondrial permeability transition regulates mitochondrial function. Curr. Pharm. Des. 29 (8), 620–629. 10.2174/1381612829666230313111314 36915987

